# Pentadecapeptide BPC 157 Reduces Bleeding and Thrombocytopenia after Amputation in Rats Treated with Heparin, Warfarin, L-NAME and L-Arginine

**DOI:** 10.1371/journal.pone.0123454

**Published:** 2015-04-21

**Authors:** Mirjana Stupnisek, Antonio Kokot, Domagoj Drmic, Masa Hrelec Patrlj, Anita Zenko Sever, Danijela Kolenc, Bozo Radic, Jelena Suran, Davor Bojic, Aleksandar Vcev, Sven Seiwerth, Predrag Sikiric

**Affiliations:** 1 Faculty of Medicine, J.J. Strossmayer University of Osijek, Osijek, Croatia; 2 Department of Pharmacology, School of Medicine, University of Zagreb, Zagreb, Croatia; 3 Department of Pathology, School of Medicine, University of Zagreb, Zagreb, Croatia; Yale University School of Medicine, UNITED STATES

## Abstract

**Background:**

BPC 157 is a stable gastric pentadecapeptide recently implicated with a role in hemostasis. While NO is largely implicated in hemostatic mechanisms, in tail-amputation-models under heparin- and warfarin-administration, both the NO-synthase (NOS)-blocker, L-NAME (prothrombotic) and the NOS-substrate L-arginine (antithrombotic), were little investigated. Objective. To investigate the effect of L-NAME and L-arginine on hemostatic parameters, and to reveal the effects of BPC 157 on the L-NAME- and L-arginine-induced hemostatic actions under different pathological condition: tail amputation without or with anticoagulants, heparin or warfarin.

**Methods:**

Tail amputation, and/or i.v.-heparin (10 mg/kg), i.g.-warfarin (1.5 mg/kg/day for 3 days) were used in rats. Treatment includes BPC 157, L-NAME, L-arginine, per se and their combination.

**Results:**

After (tail) amputation, with or without i.v.-heparin or i.g.-warfarin, BPC 157 (10 μg/kg, 10 ng/kg, i.p., i.v. (heparin), 10 μg/kg i.g. (warfarin)) always reduced bleeding time and/or haemorrhage and counteracted thrombocytopenia. As for L-NAME and/or L-arginine, we noted: L-arginine (100 mg/kg i.p.)–rats: more bleeding, less/no thrombocytopenia; L-NAME (5 mg/kg i.p.)-rats: less bleeding (amputation only), but present thrombocytopenia; L-NAME+L-arginine-rats also exhibited thrombocytopenia: L-NAME counteracted L-arginine-increased bleeding, L-arginine did not counteract L-NAME-thrombocytopenia. All animals receiving BPC 157 in addition (BPC 157μg+L-NAME; BPC 157μg+L-arginine, BPC 157μg+L-NAME+L-arginine), exhibited decreased haemorrhage and markedly counteracted thrombocytopenia.

**Conclusions:**

L-NAME (thrombocytopenia), L-arginine (increased haemorrhage) counteraction and BPC 157 (decreased haemorrhage, counteracted thrombocytopenia) with rescue against two different anticoagulants, implicate a BPC 157 modulatory and balancing role with rescued NO-hemostatic mechanisms.

## Introduction

This study further extends and clarifies the effect of the stable gastric pentadecapeptide BPC 157 on hemostasis [1,2] with addition of N(G)-nitro-L-arginine methylester (L-NAME) and L-arginine and heparin and warfarin in rats.

NO is an endogenously produced vasodilator and is largely implicated in hemostatic mechanisms [3–9] while, in tail amputation and heparin-/warfarin-administration models, both the NO-synthase (NOS)-blocker, L-NAME (thought to have a prothrombotic effect) [3] and NOS-substrate, L-arginine [presented with an antithrombotic effect) [3] were little investigated. The simultaneous counteraction of these two NO-mediated opposite effects in vivo has never been addressed.

To counteract the consequences of L-NAME and/or L-arginine administration with the disturbed hemostasis, we administered the stable gastric pentadecapeptide BPC 157 [1,2,10–26] not only due to its special effect on hemostasis [1,2], but also owing to a particular beneficial combining [1,2,10–14], wound healing capacity [1,2,10–17] and particular interaction with NO-system [14,15,18–26]. Previously, BPC 157 as an originally anti-ulcer peptide was implemented in inflammatory bowel disease trials [10–15], and now multiple sclerosis [16], lethal dose (LD1) could be not achieved [10–16]. As mentioned, this unusual combining effect in hemostasis was recently evidenced with bleeding/thrombocytopenia after amputation, with or without anticoagulant and aspirin administration, abdominal aorta anastomotic site-thrombosis, both being counteracted [1,2]. Thereby, a particular cluster of effects on the events ensuing after loss of vascular integrity, is evident [1,2,14,15].

Wound healing capacity in different tissues [1,2,10–17] (including blood vessels) (i.e., stimulation of the early growth response 1 (egr-1) gene and its co-repressor nerve growth factor 1-A binding protein-2 (naB2) [17] also responsible for cytokine and growth factor generation and thereby, early extracellular matrix (collagen) and blood vessel formation [17]) and endothelium protection [1,2,14,15] were implicated in these counteracting effects.

Also, interactions with NO-system are shown in different models and various species [12,13,18–26]. As an illustrative analogy for counteraction of the opposite effects of L-NAME and L-arginine in haemorrhage, we should emphasize that the particular effects of BPC 157 on blood pressure [18,20,25] and the anti-arrhythmic effect [18–21] include antagonization of both L-NAME (hypertension) and L-arginine (hypotension) [25] and counteraction of endothelin serum increase [20]. Note, endothelin per se, inhibited platelet aggregation and caused subarachnoid hemorrhage [27,28].

Thus, it was likely that after tail amputation in rats, with/out heparin and warfarin administration, BPC 157 would counteract the consequences of administration of the NOS-blocker, L-NAME (i.e., prothrombotic effect and thereby thrombocytopenia) and/or NOS-substrate, L-arginine (i.e., antithrombotic effect and thereby more bleeding) [29–31].

## Materials and Methods

### Animals

Male Albino Wistar rats were used in all of the experiments (10 rats per experimental group and interval). The study was approved by the Local Ethics Committee at School of Medicine (University of Zagreb, Zagreb, Croatia) and experiments were assessed by observers unaware of the given treatment.

### Drugs

As previously [1,14,15] medication, without carrier or peptidase inhibitor, included stable gastric pentadecapeptide BPC 157 (a partial sequence of the human gastric juice protein BPC, freely soluble in water at pH 7.0 and in saline). It was prepared as a peptide with 99% (HPLC) purity (1-des-Gly peptide was the main impurity; manufactured by Diagen, Ljubljana, Slovenia, GEPPPGKPADDAGLV, M.W. 1419) (in dose and application regimens as described before [1,14,15]. Likewise, in dose and application regimens as described before heparin (Belupo, Croatia), warfarin (Martefarin (Orion Pharma, Finland)), L-NAME (Sigma, USA) and L-arginine (Sigma, USA) dissolved in saline were used as well [1,14,15].

### Bleeding procedures, heparin, warfarin application and medication

Bleeding was monitored as described before [1], until blood flow stopped for a complete 30-s interval or till the end of 60 min period. In deeply anaesthetised rats (placed in ventral position) the tail was transected with a surgical scalpel 3 cm from the tip (and submerged into a tube with 15 ml of saline at room temperature in vertical position) and the duration and amount of bleeding were measured to evaluate the hemostatic effect of the agents or saline administration. The blood samples assessment was carried precisely as described before [1] (Table 1).

**Table 1:** The blood samples assessment, laboratory methods used in the study.

Parameters	Methods/Reagents	Optimum specimens/ Tested within	Analyzers	Units
Prothrombin time (PT)	Performed according to the technique by Quick using human placental thromboplastin (Thromborel S, Siemens Healthcare Diagnostics Products GmbH, Marburg, Germany)	Plasma/ 2 hours	Automated coagulation analyzer BCS XP System (Siemens, Marburg, Germany)	ratio
Activated partial thromboplastin time (APTT)	Performed by using Actin FS (Dade Actin FS Activated PTT Reagent, Siemens Healthcare Diagnostics Products GmbH, Marburg, Germany) containing the purified soy phosphatides	Plasma/ 2 hours	Automated coagulation analyzer BCS XP System (Siemens, Marburg, Germany)	ratio
Thrombin time (TT)	Performed according to the instructions from manufacturer using bovine thrombin (BC Thrombin Reagent, Siemens Healthcare Diagnostics Products GmbH, Marburg, Germany)	Plasma/ 2 hours	Automated coagulation analyzer BCS XP System (Siemens, Marburg, Germany)	sec
Fibrinogen concentration (FIB)	Determinated by a modification of Clauss' coagulometric method using bovine thrombin (Multifibren U, Siemens Healthcare Diagnostics Products GmbH, Marburg, Germany)	Plasma/ 2 hours	Automated coagulation analyzer BCS XP System (Siemens, Marburg, Germany)	g/L
Platelet count (PLT)	Performed by using Coulter principle (impedance) (Beckman Coulter Reagents)	Whole blood/ 2 hours	Automated Coulter HmX Hematology Analyzer (Beckman Coulter Inc., Florida, USA)	10^9^/L
Hematocrit (HTC)	Performed by directly measuring from RBC histogram	Whole blood/ 2 hours	Automated Coulter HmX Hematology Analyzer (Beckman Coulter Inc., Florida, USA)	L/L

In addition, platelet counts were corrected with hematocrit to avoid errors that may be caused by animals bleeding, and were expressed as a percentage of baseline for each time point of the tested groups. To obtain the hematocrit-corrected platelet count (X) we used the following formula: X = (h1xp1 / HxP) x 100, wherein each symbol denotes the following: h1: hematocrit of the sample, p1: platelet count of the sample, H: mean hematocrit value of the normal range in healthy animals (= 0.435), P: mean platelet count value of the normal range in healthy animals (= 875). HxP result was considered as a baseline of 100%.

Finally, using our own research values determined in healthy rats, prothrombin time (PT) ratio 1.25–1.45, activated partial thromboplastin time (APTT) ratio 0.38–0.51, thrombin time (TT) 30–50 sec, fibrinogen concentration (FIB) 1.5–2.0 g/L, platelet count (PLT) 650–1100 x10^9^/L, hematocrit (HTC) 0.38–0.49 L/L were considered as the normal range [1].

### Amputation only

BPC 157 (10 μg/kg, 10 ng/kg), L-NAME (5 mg/kg), L-arginine (100 mg/kg), given alone or combined, were applied intraperitoneally, at 30 minutes before amputation while controls received simultaneously an equivolume of saline (5ml/kg intraperitoneally).

### Amputation associated with heparin administration

Heparin (10 mg/kg) was given intravenously at 30 minutes before the bleeding procedure. As a medication, we applied BPC 157 (10 μg/kg, 10 ng/kg), L-NAME (5 mg/kg), L-arginine (100 mg/kg) alone and/or combined, intravenously, immediately after intravenous heparin administration, while controls received simultaneously an equivolume of saline (0.5 ml/kg intravenously).

### Amputation associated with warfarin administration

Warfarin was given intragastrically (1.5 mg/kg) once daily for 3 consecutive days, with the last challenge at 3 hours before the bleeding procedure. BPC 157 (10 μg/kg, 10 ng/kg) was given immediately after any warfarin challenge, intragastrically while controls received simultaneously an equivolume of saline (5.0 ml/kg intragastrically). We applied L-NAME (5 mg/kg), L-arginine (100 mg/kg) given per se or combined with each other, intraperitoneally, at 30 minutes before amputation.

### Statistical analyses

Statistical analyses of the quantified data were performed by analysis of variance (ANOVA). Post-hoc comparisons were appraised using the conservative Bonferroni/Dunn test. Data are presented as the mean ± standard deviation (SD). Values of P<0.05 were considered statistically significant.

## Results

**Fig 1:**
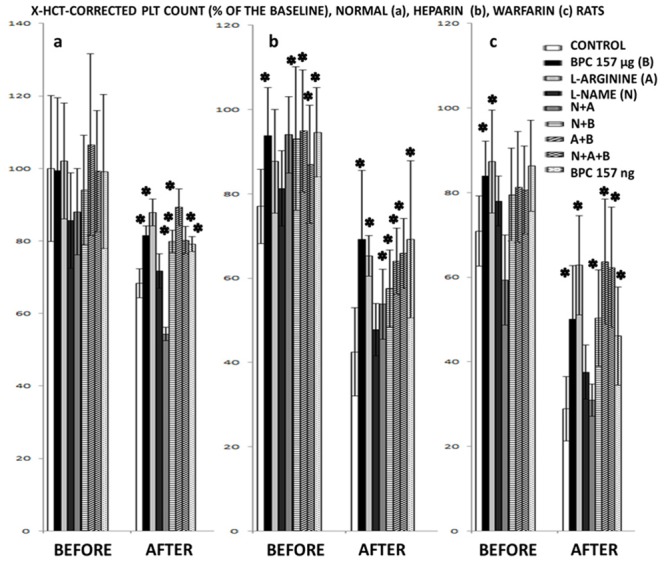
Hematocrit-corrected platelet counts (X) before, and after bleeding period. Hematocrit-corrected platelet counts (X) before, and after bleeding period: (a) in normal rats, BPC 157 (10 μg/kg, 10 ng/kg), L-NAME (5 mg/kg), L-arginine (100 mg/kg) given alone and/or together,applied intraperitoneally, at 30 minutes before amputation while controls received simultaneously an equivolume of saline (5ml/kg intraperitoneally); (b) in heparin (10 mg/kg intravenously) challenged rats when treated with BPC 157 (10 μg/kg, 10 ng/kg), L-NAME (5 mg/kg), L-arginine (100 mg/kg) alone or combined, intravenously, immediately after intravenous heparin while controls received simultaneously an equivolume of saline (0.5 ml/kg intravenously); (c) in warfarin rats (warfarin given intragastrically (1.5 mg/kg) once daily for 3 consecutive days, with the last challenge was at 3 hours before the bleeding procedure). BPC 157 (10 μg/kg, 10 ng/kg) was given immediately after any warfarin challenge, intragastrically while controls received simultaneously an equivolume of saline (5.0 ml/kg intragastrically). We applied L-NAME (5 mg/kg), L-arginine (100 mg/kg) alone or combined, intraperitoneally, at 30 minutes before amputation. Mean ± SD, *P<0.05 at least vs. control, 10 rats at least per group.

Here, in normal rats, after tail amputation (spontaneous bleeding for 20 minutes, fall in platelet count without any failure of coagulation parameters) the previous effects of BPC 157 (reduced bleeding, no thrombocytopenia) [15] were confronted with that of L-arginine (prolonged bleeding without thrombocytopenia) and L-NAME (reduced bleeding, thrombocytopenia present), given alone and/or combined. Then, these effects were confronted with administration of anticoagulants without or with amputation; heparin (extensive bleeding and blood loss, prominent fall in platelet count, drastic prolongation of PT-, APTT-, TT- values) or warfarin (extensive bleeding and blood loss, prominent fall in platelet count, drastic prolongation of PT-, APTT- values) (Table 2, Table 3 and Table 4). These effects were specifically assessed as follows (in addition, platelet counts were additionally corrected with hematocrit to avoid errors that may be caused by animals bleeding (Fig 1)).

**Table 2:** Normal rats.

Tail amputation	Bleeding time	Amount of bleeding	PLT before	PLT after	HCT before	HCT after	PT	APTT	TT	FIB
	min	mL	x10^9^/L	x10^9^/L	L/L	L/L	ratio (PR)	ratio	sec	g/L
Control	18±3	1.92±1.1	853±173	605±35	0.446±0.03	0.430±0.04	1.30±0.22	0.45± 0.16	34± 12	1.6± 0.22
BPC ug	7±2*	0.21±0.06*	820±167	685± 22*	0.461±0.03	0.453±0.03	1.32±0.25	0.41± 0.17	40± 11	1.8± 0.17
BPC ng	8±2*	0.24±0.04*	831±178	675± 17*	0.454±0.04	0.446±0.03	1.35±0.21	0.42± 0.11	41± 13	1.7± 0.19
L-arginine	29±4*	1.63±1.2	781±123	702± 30*	0.497±0.03	0.476±0.03	1.29±0.21	0.64± 0.18	35± 9	1.8± 0.23
L-NAME	7±2*	0.75±0.15*	677± 105*	601±40	0.481±0.04	0.454±0.03	1.28±0.19	0.66± 0.21	30± 8	1.8± 0.18
L-NAME+ L-arginine	21±3	0.8±0.24*	695±94*	442± 15*	0.482±0.05	0.468±0.03	1.24±0.22	0.51± 0.13	46± 14	1.8± 0.21
L-arginine+BPC 157 ug	9±2*	0.57±0.11*	801±189	694± 39*	0.506±0.04	0.490±0.04	1.28±0.21	0.49± 0.17	39± 11	1.8± 0.27
L-NAME+ BPC 157 ug	8±2*	0.40±0.12*	758±122	654± 25*	0.472±0.03	0.465±0.04	1.30±0.22	0.49± 0.18	40± 10	1.8± 0.19
L-NAME+L-arginine+ BPC 157 ug	10±3*	0.30±0.09*	788±133	655± 31*	0.479±0.03	0.466±0.03	1.34±0.23	0.5± 0.13	48± 13	1.8± 0.18

Tail amputation, bleeding time, amount of bleeding, PLT, HCT, PT, APTT, TT, FIB, values in normal rats (10 rats at least per group) with amputation challenged with BPC 157 (10 μg/kg, 10 ng/kg), L-NAME (5 mg/kg), L-arginine (100 mg/kg) given alone and/or together,applied intraperitoneally, at 30 minutes before amputation while controls received simultaneously an equivolume of saline (5ml/kg intraperitoneally). Mean ± SD,

*P<0.05 at least vs. control.

**Table 3:** Heparin rats.

Heparin 10 mg/kg i.v.	Bleeding time	Amount of bleeding	PLT before	PLT after	HCT before	HCT after	PT	APTT	TT	FIB
Tail amputation	min	mL	x10^9^/L	x10^9^/L	L/L	L/L	ratio (PR)	ratio	sec	g/L
Control	>60	8.82± 2.2	654± 75	426± 105	0.448± 0.02	0.380+ 0.03	0.87±0.15	4.07± 1.21	>150	1.5± 0.24
BPC ug	31± 8*	5.08± 1.3*	768± 93*	600± 122*	0.465± 0.03	0.439+ 0.03*	0.91±0.17	2.46± 1.16*	91± 14*	1.6± 0.17
BPC ng	35± 9*	5.45± 1.1*	788± 88*	611± 133*	0.457± 0.03	0.434+ 0.04*	0.93±0.20	2.00± 1.05*	101± 21*	1.5± 0.21
L-arginine	>60	11.5± 3.4*	784± 110*	698± 51*	0.426± 0.02	0.356± 0.03	0.33± 0.06	>5.7	>150	1.1± 0.20
L-NAME	>60	8.0± 2.1	727± 80*	479± 62	0.426± 0.04	0.380± 0.03	0.35± 0.08	>5.7	>150	1.2 ± 0.19
L-NAME+ L-arginine	>60	8.10± 2.8	819± 79*	498± 77	0.437± 0.03	0.411± 0.04	0.39±0.09	>5.7	>150	1.0± 0.15
L-arginine+ BPC 157 ug	>60	7.3± 1.5*	852± 130*	721± 89*	0.424± 0.04	0.338± 0.02	0.38±0.08	>5.7	>150	1.4± 0.16
L-NAME+ BPC 157 ug	>60	5.5± 1.3*	844± 155*	601± 95*	0.420± 0.03	0.364± 0.03	0.35±0.07	>5.7	>150	1.3± 0.16
L-NAME+L-arginine+ BPC 157 ug	>60	5.1± 1.4*	800± 129*	658± 83*	0.414± 0.05	0.381± 0.03	0.41±0.10	>5.7	>150	1.2± 0.16

Tail amputation, bleeding time, amount of bleeding, PLT, HCT, PT, APTT, TT, FIB values in heparin (10 mg/kg intravenously) challenged rats (10 rats at least per group) when treated with BPC 157 (10 μg/kg, 10 ng/kg), L-NAME (5 mg/kg), L-arginine (100 mg/kg) alone or combined, intravenously, immediately after intravenous heparin while controls received simultaneously an equivolume of saline (0.5 ml/kg intravenously). Mean ± SD,

*P<0.05 at least vs. control.

**Table 4:** Warfarin rats.

Warfarin 1.5 mg/kg i.g. once daily for 3 consecutive days	Bleeding time	Amount of bleeding	PLT before	PLT after	HCT before	HCT after	PT	APTT	TT	FIB
Tail amputation	min	mL	x10^9^/L	x10^9^/L	L/L	L/L	ratio (PR)	ratio	sec	g/L
Control	>60	6.58± 1.2	612± 72	302± 79	0.441± 0.04	0.364± 0.02	<0.05	2.86± 0.42	62± 6	1.5± 0.15
BPC ug	48.6± 15*	5.52± 1.4*	693± 68*	456± 116*	0.461± 0.03	0.417± 0.02*	<0.05	2.81± 0.38	60± 7	1.7± 0.25
BPC ng	>60	5.65± 1.1*	706± 88*	418± 105*	0.465± 0.02	0.420± 0.03*	<0.05	2.83± 0.47	58± 8	1.5± 0.21
L-arginine	>60	7.8± 1.4	638± 89	525± 98*	0.521± 0.06	0.455± 0.04	<0.05	2.03± 0.36	59.9± 8	1.9± 0.19
L-NAME	>60	8.0± 1.8	654± 49	346± 59	0.454± 0.03	0.413± 0.03	<0.05	2.60± 0.39	62.6± 7	1.8± 0.17
L-NAME+ L-arginine	>60	7.0± 1.7	483± 87*	283± 35	0.467± 0.04	0.415± 0.03	<0.05	2.68± 0.34	60.6± 8	1.8± 0.21
L-arginine+ BPC 157 ug	>60	4.9± 0.6*	650± 105	514± 119*	0.476± 0.04	0.472± 0.04	<0.05	2.03± 0.38	59.8± 8	1.6± 0.17
L-NAME+ BPC 157 ug	>60	5.0± 0.9*	637± 88	461± 105*	0.475± 0.04	0.415± 0.02	<0.05	1.74± 0.29	60.1± 7	1.6± 0.15
L-NAME+L-arginine+ BPC 157 ug	>60	4.5± 0.7*	612± 79	493± 113*	0.501± 0.05	0.481± 0.03	<0.05	1.92± 0.31	78.3± 12	1.8± 0.18

Tail amputation, bleeding time, amount of bleeding, PLT, HCT, PT, APTT, TT, FIB values in warfarin rats. Warfarin was given intragastrically (1.5 mg/kg) once daily for 3 consecutive days, with the last challenge was at 3 hours before the bleeding procedure. BPC 157 (10 μg/kg, 10 ng/kg) was given immediately after any warfarin challenge, intragastrically while controls received simultaneously an equivolume of saline (5.0 ml/kg intragastrically). We applied L-NAME (5 mg/kg), L-arginine (100 mg/kg) alone or combined, intraperitoneally, at 30 minutes before amputation. Mean ± SD,

*P<0.05 at least vs. control.

### BPC 157-rats

Specifically, BPC 157, 10 μg/kg, 10 ng/kg regimens, exhibited a consistent counteracting effect (i.e., markedly reduced the bleeding time, blood loss and also counteracted: acute thrombocytopenia in rats with amputation only, in heparin-rats or warfarin-rats), prolonged APTT-, TT- values (heparin-rats), but not PT-values (warfarin-rats)).

### L-arginine-rats, L-NAME-rats, L-NAME+L-arginine-rats

Alone, L-arginine prolonged bleeding time (rats with amputation only) and increased the amount of bleeding (heparin-rats, warfarin-rats) while L-NAME reduced bleeding time (rats with amputation only). L-NAME+L-arginine (combined) exhibited a decrease in bleeding amount and laboratory values in comparison to controls (amputation only) or control-heparin and control-warfarin groups. Commonly, controls (amputation only, heparin-rats and warfarin-rats) exhibited thrombocytopenia. L-arginine counteracted thrombocytopenia (L-arginine-rats with amputation only and L-arginine-heparin-rats are without thrombocytopenia, L-arginine-warfarin-rats are with thrombocytopenia). Contrary to this, L-NAME-rats or L-NAME+L-arginine-rats consistently exhibited thrombocytopenia.

BPC 157+L-arginine, BPC 157+L-NAME, BPC 157+L-arginine+L-NAME

### BPC 157+L-arginine-rats

BPC 157+L-arginine-rats exhibited shorter bleeding times than corresponding controls after amputation only; amount of bleeding being even less than that of control-heparin-rats or control-warfarin-rats, in addition to attenuated thrombocytopenia after warfarin administration and counteracted thrombocytopenia after amputation only, or amputation and heparin- or warfarin-administration.

### BPC 157+L-NAME rats

BPC 157+L-NAME rats exhibited shorter bleeding times with lesser bleeding amounts than corresponding controls after amputation only; consistently no thrombocytopenia (amputation only) or attenuated thrombocytopenia in heparin- and warfarin-rats, along with lesser bleeding amounts than corresponding control-heparin- and control-warfarin-rats.

### BPC 157+L-NAME+L-arginine-rats

BPC 157+L-NAME+L-arginine-rats after amputation only, or heparin or warfarin administration, exhibited a consistent attenuation of thrombocytopenia, along with lesser bleeding time and/or amount, than corresponding controls.

On the other hand, unlike in BPC 157-rats (BPC 157 given alone), PT-values remained prolonged (warfarin-rats), here, after heparin, or warfarin, BPC 157+L-arginine-rats, BPC 157+L-NAME-rats, BPC 157+L-arginine+L-NAME-rats exhibited no reduced bleeding time and no effect on prolonged PT-, APTT-, TT- values (heparin-rats).

## Discussion

BPC 157 has been shown to exert beneficial effects against different pathological conditions [1,2,10–26], it induced gastroprotective, analgesic and anti-inflammatory wound healing effects [1,2,10–17], also effective against experimental colitis [10–16] and showing a particular interaction with the NO-system [14,15,18–26]. Recently, we demonstrated the effect of BPC 157 against bleeding/thrombocytopenia after amputation, with or without anticoagulant and aspirin administration and abdominal aorta anastomotic site-thrombosis [1,2].

Thus, as a proper extension to the disturbed NO-system in hemostasis [3–9], the present work showed that L-NAME-induced thrombocytopenia and L-arginine-induced increased hemorrhage. These were counteracted by BPC 157, indicating the modulatory and balancing role of BPC 157 with rescued NO-hemostatic mechanisms.

Also, for the consistently reduced amount of bleeding in BPC 157-rats [1,2], the illustrative insight might be the reduced bleeding time evidence, affected by platelet function. Thus, resolving a hemostatic defect, the hallmark of which is a markedly prolonged bleeding time, BPC 157 reduced the bleeding times in the tail amputation model, heparin and partly warfarin rats, but unlike in normal rats, did not reduce bleeding time in heparin and warfarin rats when previously L-arginine or L-NAME was given. Therefore, with respect to BPC 157, L-arginine and L-NAME, heparin and warfarin, we demonstrated a complex and particular interaction.

In the normal rats, L-NAME reduced bleeding time (and induced thrombocytopenia and thereby, the NOS-blocker-prothrombotic effect), L-arginine prolonged bleeding time (thereby showing the NOS-substrate-antithrombotic effect) [29–31]. Consequently, changing these effects, we likely revealed, a particular action of L-arginine and a particular action of L-NAME on heparin and warfarin, thus far not demonstrated in amputation rat models. With respect to BPC 157, they might both augment heparin and warfarin effects and thereby BPC 157 would have a changed effect (however, as emphasized, BPC 157 always reduced the amount of bleeding). Or, more likely, it could be that their effects are both interfering with that of BPC 157. Likely, this might also be the supportive part to the general interaction. Namely, very similar interactions of BPC 157 and the NO system, with both L-NAME and L-arginine, have been demonstrated by a large number of other previous studies [14,15,18–26]. For instance, counteraction of both the L-arginine-induced disturbance and L-NAME-induced disturbance was seen, while maintaining blood pressure against both L-arginine (hypotension) and L-NAME (hypertension) (note, BPC 157 by itself does not affect basal blood pressure values [25] and likewise, BPC 157 by itself does not affect basal coagulation/platelets values [1]). Thus, this analogy might be the principal advantage for this and previous studies [14,15,18–26], with a challenge in methodology and in general, since in other studies (not related to this model) L-NAME-thrombocytopenia antagonization was not attempted [3–9,32], nor were L-arginine and L-NAME simultaneously investigated.

Furthermore, we could argue that with the prolonged bleeding time, specifically disturbed coagulation factors, with heparin (less) and warfarin (more), would affect BPC 157, L-arginine and L-NAME. Or, more likely, their effects are in general more resistant to that of heparin and less to that of warfarin, regardless of the more haemorrhage in heparin rats.

Illustratively, heparin still induced the full effectiveness of BPC 157 in both doses of application (μg, ng); thrombocytopenia was present only with L-NAME. L-arginine rats did not exhibit thrombocytopenia.

Contrary to this, particular with warfarin are: a lesser effectiveness of BPC 157 (smaller dose not effective on bleeding time, but still affects amount of bleeding and thrombocytopenia); not only with L-NAME-rats, but also those treated with L-arginine there was an initial marginal thrombocytopenia up to a very prominent thrombocytopenia after amputation, and both exhibited unaffected warfarin-bleeding amounts. Thus, with warfarin, thrombocytopenia is the common end result of the L-NAME and/or L-arginine administration. In consequence, with the thrombocytopenia and haemorrhage, the expected lack of NO-suppression of platelet aggregation [33], the acceleration and amplification of the induced pattern of platelet activation resulting in platelet exhaustion [9] (L-NAME), grossly correspond to the suggested inhibition of platelet aggregation, by direct action on an intraplatelet constitutive calcium-dependent NOS [33–35] (L-arginine). In either case, particularly considering the sustained warfarin bleeding, even when L-arginine actually attenuated thrombocytopenia, the disturbed NO-hemostatic system [27,30,31] is the entity which has to be further rescued. Thereby, it is important that thrombocytopenia appeared also in warfarin L-NAME+L-arginine-rats and that it is attenuated by the addition of BPC 157 (L-NAME+L-arginine+BPC 157-rats) and reduced the amount of bleeding. Likewise, the addition of BPC 157 to the L-NAME (L-NAME+BPC 157-rats) or to the L-arginine (L-arginine+BPC 157-rats), equally reduced the amount of bleeding. Thereby, we argue that the better hemostasis was achieved with BPC 157 administration, the balanced role of the L-arginine and of the L-NAME and the more rescued NO-hemostatic mechanisms.

The supportive argument may be that BPC 157 by itself induces NO-release (demonstrated from gastric mucosa supernatant), like L-arginine, but also in conditions where L-arginine is not working [14].

Consequently, whatever the fundamental mechanism of BPC 157 beneficial effects, there was a BPC 157-induced counteraction of the two different anticoagulants and of the L-NAME and the L-arginine. Together, these counteractions could obviously suggest an alternative common pathway.

Possibly, the consistent endothelium protection during advanced healing processes in different wounds of various tissues, including blood vessels [1,2,10–17], associated with the counteraction of the L-NAME-induced disturbances as well as the counteraction of the L-arginine-induced disturbances, obtained with the same BPC 157 dose range [14,15,18–26], should likely compete with the prominent endothelium effect of the heparin-impaired endothelial nitric oxide (NO) synthesis [36] as well as warfarin endothelium damage [37]. Finally, as emphasized, BPC 157 beneficial effects include stimulation of egr-1 gene and its co-repressor gene naB2 [17], also responsible for cytokine and growth factor generation and thereby, early extracellular matrix (collagen) and blood vessel formation [17]. Egr-1 functions as a master switch activated by ischemia to trigger expression of pivotal regulators of inflammation, coagulation and vascular hyperpermeability [38]. Thus, BPC 157 by naB2 regulation could represent a mechanism to guarantee a suited transient EGR-1 activity following injury [39]. Supporting may also be the BPC 157-induced decrease of the inflammatory mediators, leukotriene B4 (LTB4), thromboxane B2 (TXB2), and myeloperoxidase (MPO), in both serum and inflamed tissue [40], largely implicated in heparin-induced platelet activation, the adhesion of leukocytes to the endothelium and concomitant increase in vascular permeability [41], prolongation of the bleeding time [42] while low-molecular-weight heparin enoxaparin causes systemic MPO activation [43]. Lastly, implicated in coagulation disorders as well as in anaphylaxis [44], in response to stimuli such inflammation and trauma, mast cells degranulate and consequently release heparin [45] while BPC 157 pretreatment significantly prevented mastocyte infiltration [46]. Also, BPC 157 rescued already advanced anaphylactoid reactions caused by mastocytes degranulation when given after the challenge [47].

In conclusion, these results accord with the previous NO-system studies [10–26] and the particular BPC 157 effect on hemostasis [1,2]. Along with the BPC 157 safety evidence (LD1 could be not achieved, and without side effects in patients and healthy subjects) [10–16], these likely suggest that BPC 157 could solve the lack of specific antagonist as a major concern associated with the anticoagulant clinical use.
